# Laser Scribing Turns Plastic Waste into a Biosensor via the Restructuration of Nanocarbon Composites for Noninvasive Dopamine Detection

**DOI:** 10.3390/bios13080810

**Published:** 2023-08-12

**Authors:** Jagadeesh Suriyaprakash, Yang Huang, Zhifei Hu, Hao Wang, Yiyu Zhan, Yangtao Zhou, Indumathi Thangavelu, Lijun Wu

**Affiliations:** 1 Guangdong Provincial Key Laboratory of Nanophotonic Functional Materials and Devices, School of Information and Optoelectronic Science and Engineering, South China Normal University, Guangzhou 510006, China; jaga@m.scnu.edu.cn (J.S.); 2022022436@m.scnu.edu.cn (Y.H.); 20193231019@m.scnu.edu.cn (Z.H.); 2021022483@m.scnu.edu.cn (H.W.); 2022022421@m.scnu.edu.cn (Y.Z.); 2 Shenyang National Laboratory for Materials Science, Institute of Metal Research, Chinese Academy of Sciences, Wenhua Road 72, Shenyang 110016, China; ytzhou@imr.ac.cn; 3 Department of Chemistry, CHRIST (Deemed to be University), Bangalore 560029, Karnataka, India; indumathi.t@christuniversity.in

**Keywords:** flexible sensor, direct laser writing, carbon materials, dopamine, biosensor, electrochemical

## Abstract

The development of affordable and compact noninvasive point-of-care (POC) dopamine biosensors for the next generation is currently a major and challenging problem. In this context, a highly sensitive, selective, and low-cost sensing probe is developed by a simple one-step laser-scribing process of plastic waste. A flexible POC device is developed as a prototype and shows a highly specific response to dopamine in the real sample (urine) as low as 100 pmol/L in a broad linear range of 10^−10^–10^−4^ mol/L. The 3D topological feature, carrier kinetics, and surface chemistry are found to improve with the formation of high-density metal-embedded graphene-foam composite driven by laser irradiation on the plastic-waste surface. The development of various kinds of flexible and tunable biosensors by plastic waste is now possible thanks to the success of this simple, but effective, laser-scribing technique, which is capable of modifying the matrix’s electronic and chemical composition.

## 1. Introduction

The most significant neurotransmitter that influences human behavior, as well as vital physiological processes in the human body, is dopamine (DA) [1,2]. Our body’s DA imbalance causes severe mental issues as well as other ailments including Alzheimer’s disease, Parkinson’s disease, obesity, and attention deficit hyperactivity disorder (ADHD), especially in children [3,4,5,6,7]. Thus, continuous monitoring of this neurotransmitter is necessary in order to perform early diagnosis and offer suitable medical care to the user end. Noninvasive methods should be considered as one of the most effective ways to continuously monitor DA. In general, DA is present in the urine of healthy people at very low concentrations ranging from 274 to 500 nmol/L, with potential interferents, and concentrations less or higher than this range are considered to be a DA imbalance (for unhealthy people) over a 24 h period [8]. Regrettably, there are very few methods available for the detection of DA at ultralow levels. These analytical procedures depend on costly instruments, are time-consuming, and need a skilled person for precise preparation of the sampling, which makes noninvasive DA detection for point of care (POC) continuous monitoring extremely difficult.

Recent studies demonstrate that electrochemical sensing is one of the most promising methods for determining neurotransmitters at the pmol/L level in biofluids and designing POC devices [9,10,11,12,13]. However, due to the utilization of costly metals [14,15,16,17,18,19], aptamers [20,21], processed nanocarbon [22,23,24,25], enzymes [9,26], hybrid composites [27,28,29,30,31,32,33,34,35,36,37,38,39,40,41,42,43], and complicated fabrication process [44,45], their application for creating large-scale recognizing units/electrodes is still restricted, yet they offer good sensitivity. Therefore, a novel sensor that is highly efficient and economical must be designed without a complex manufacturing technique. In this context, researchers have recently focused on laser-induced graphene (LIG) composites due to their reasonable conductivity, electrocatalytic nature, and the chemical-free flexible fabrication process for the easy integration of electrodes. However, LIG is mostly explored in dense commercial polymers [46,47,48,49,50,51,52,53,54,55], which have limitations in terms of in situ functionalization, flexibility, transparency, and so on. In light of these constraints, the exploration of this research field is currently wide open and there is a strong desire to develop next-generation noninvasive electrochemical biosensors.

Herein, we develop a flexible and highly effective DA sensor through a simple one-step laser-scribing process of plastic/polymer waste. The newly developed sensor consists of a laser-induced metal-embedded graphene nanocomposite (LIMG), which has extraordinary electrocatalytic activity, flexibility, and stability. A unique high-density metal-embedded graphene-foam material is generated when the surface of metal-ion-incorporated plastic waste (Polyethylene terephthalate [PET]) is exposed to laser radiation under appropriate conditions. A flexible and portable POC device is built as a proof of concept. The sensor is capable of detecting DA as low as 100 pmol/L in human urine samples. This study is the first example of electrochemical DA detection in a real biological sample noninvasively using plastic waste as a basic source. This new technique facilitates the efficient conversion of plastic waste into a biosensing platform. The details of this study are provided below.

## 2. Methods

### 2.1. Sensor Fabrication

**Figure 1:**
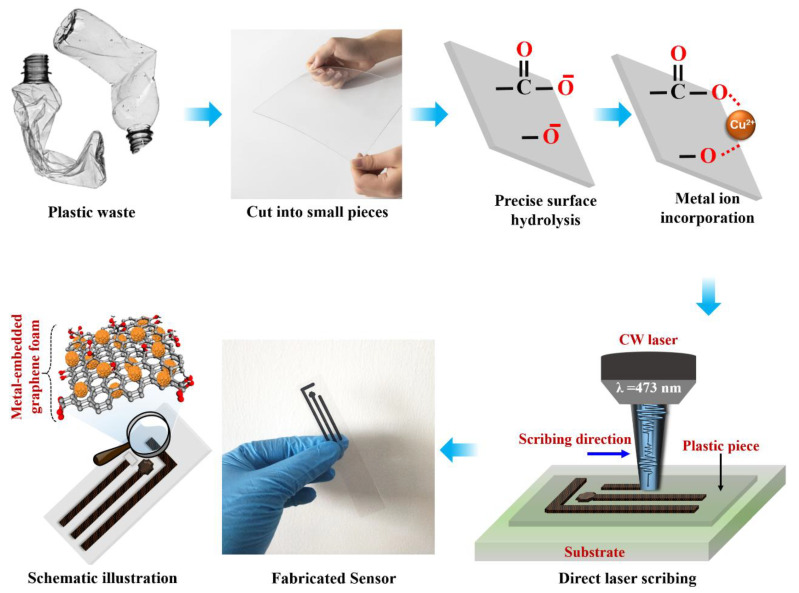
Schematic illustration of the complete fabrication process of the biosensor from plastic waste.

As illustrated in Figure 1, the fabrication setup was designed. First, waste polyethylene terephthalate (WPET) bottles were thoroughly washed with MilliQ water. They were subsequently cut into small rectangle-shaped sheets and pressed to form neat sheet shapes. The chemical composition of the waste PET bottles is subjected to FTIR spectral analysis to ensure the reliability of the source material (Figure S6). The surface of the PET sheets was precisely hydrolyzed in the second stage causing no damage to the interior structure. At 50 °C, the WPET sheets were immersed in a NaOH (pH 9) solution for 3 min. After the hydrolysis process, WPET sheets were washed, dried, and stored for further process. Afterward, a single 5 cm × 5 cm WPET sheet was placed into a 0.01 M Copper dithiocarbamate/DMSO solution for about 1 h; this process enables the metal ion incorporated into WPET’s active surface, as shown in Figure 1.

A continuous-wave (CW) laser beam at a wavelength of 473 nm was utilized as an irradiation source. After measuring the UV-Vis spectra of precursor samples in each stage (Figure S1), the laser light wavelength is selected; it is critical to select a material that can produce an appropriate photothermal effect without damaging the substrate during the laser-scribing process. To control the energy and polarization direction of the laser beam, an attenuator and a half-wave plate were used. An oil-immersion objective lens (NA = 1.4, Zeiss, Jena, Germany) was employed to focus the laser beam properly on the sample with a spot size of 200 µm. The sample was mounted on a three-dimensional high-precision nanopositioning translation stage (P-563, PI) controlled by a computer. To find out the optimized condition for laser scribing, various physicochemical parameter combinations are used (power, wavelength, Cu ion source, soaking time, etc.). Since it is outside the scope of this article, a separate research paper describing the comprehensive dedicated work of laser interaction with the surface of metal-ion polymer waste and the method of creation will be provided. The desired patterns were created by laser scribing at a power of 70.03 mJ µm^−2^ and a speed of 5 µm/s. The samples were thoroughly cleaned with MilliQ water after the laser-scribing process and, then, they were allowed to air dry out naturally. The scribed area of PET samples produced the LIMG, which was utilized for further investigation.

### 2.2. Fabrication of Flexible POC Device

The portable flexible POC sensor was fabricated by modifying a working electrode (WE) area and a reference electrode (RE) was constructed using a colloidal Ag/AgCl (60/40) paste. A 50 μL solution of 1-ethyl-3-(3-dimethylaminopropyl) carbodiimide, together with N-hydroxysuccinimide 1:1 (by volume), was drop-casted onto the WE surface and left for 10 min. Then, 10 μL of 10 units/μL tyrosinase in 50 mmol/L PBS for 6 h was added to the WE area. This procedure was repeated 2 times. Further, to remove the detached enzyme, the WE was washed in 50 mmol/L PBS (pH 7.2) solution. This process produced a tyrosinase-immobilized laser-induced metal-embedded graphene nanocomposite (Tyr/LIMG) sensor. Subsequently, the desired Tyr/LIMG sensors were then produced by adding 1 mol/L ethanolamine to immobilize the uncovered active sites. The laser-scribed pattern acted as a counter electrode (CE) and an electrical contact. To insulate the contact of the electrodes, the PI tape was laminated on top of the integrated sensor. A schematic illustration and a photographic image are presented in Figure 1, respectively, for easy understanding. The Supplementary Information contains the detailed experimental procedure.

## 3. Results and Discussion

### 3.1. Morphological, Surface, and Structural Evaluation

**Figure 2:**
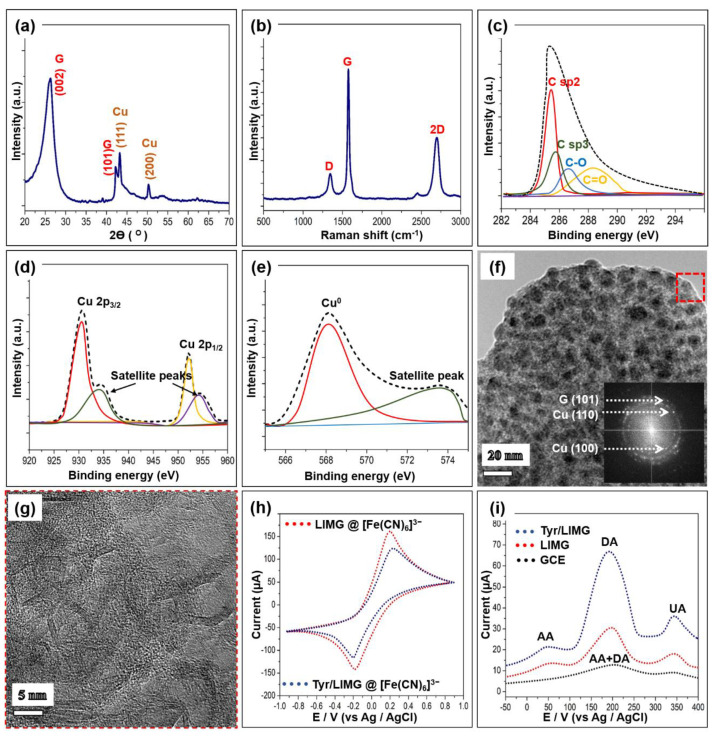
(**a**,**b**) XRD/Raman spectra of the LIMG. Deconvoluted XPS core-level spectra (**c**) Carbon, (**d**) Copper 2p, (**e**) Copper LMM Auger spectrum. (**f**) TEM image of LIMG, Inset: SAED pattern of the corresponding sample. (**g**) The HRTEM image of the selected region is denoted as a red rectangle in (**f**). (**h**) Cyclic voltammograms of the LIMG and Tyr/LIMG electrodes in [Fe(CN)_6_]^3−^/PBS solution at a scan rate of 100 mVs^−1^. (**i**) DPV results in the presence of the mixture containing 1 mM ascorbic acid, 0.1 mM dopamine, and 1 mM uric acid.

Figure 2a displays the X-ray diffraction patterns of the laser-induced structure. It exhibits well-crystalline graphitic nature peaks (denoted as G) [56] along with copper metal crystal planes [57]. The peaks (denoted as Cu) can be indexed as cubic-phase metallic copper PDF# 85-1326. There are no discernible impurity peaks, confirming that the newly formed structure is a copper–graphene-like composite. In addition, the Raman spectrum of the fabricated sample is obtained to reveal the structural evolution of the metal–graphene composite. As shown in Figure 2b, the graphene feature exhibits the typical D band (around 1341 cm^−1^), G band (around 1574 cm^−1^), and 2D band (2684 cm^−1^). The intensity ratio of the designated peaks I_D_, I_G_, and 2_D_ is quite different from that of the standard graphene feature [10]. It could be because of the strong metal–graphene interaction, where the nature of the peak is influenced by localized surface plasmon resonance (SPR) [58].

X-ray photoelectron spectroscopy (XPS) analysis is also used to investigate the surface nature of the LIMG, as shown in Figure 2c–e. The spectrum of C 2p (Figure 2c) can be deconvoluted into one major and three minor components in the 285–292 eV range. These are attributed to the presence of various chemical moieties, such as –C=O, –C–O, sp2 C–C, and sp3 C–C [9]. It implies that the laser-induced surface of plastic waste has –COOH and –COH functional groups, which enable hydrophilicity and easy linkage with other chemical species. Figure 2d displays the core-level spectrum of Cu 2p peaks showing two distinct peaks at 952 and 929 eV, which correspond to 2p_3/2_ and 2p_1/2_ along with satellite peaks (Cu 2p ions) [59]. To confirm the presence of a Cu^0^ metallic state, the Cu LMM Auger spectrum is obtained, as shown in Figure 2e. The deconvoluted spectra consist of one major and one minor peak. The major peak at 568.3 eV can be attributed to the Cu^0^ metallic species. These results evidence the existence of a Cu metallic and Cu ion type linkage formed during the fabrication process. Figure 2f is a typical TEM image of the fabricated sample that displays tiny metallic particles with an average size of 12.1 nm (Figure S1) embedded in the nanocarbon matrix. Moreover, the SAED pattern of the corresponding region reveals diffraction rings indicative of the random crystallographic orientations of the nanocrystal of the as-fabricated material. All diffraction rings are identified as that of a cubic structure of copper metal and graphitic nature, which is consistent with the XRD and XPS data. The bright spots in the ring pattern can be well indexed to the (100), (110) Cu, and (101) graphene planes. The HRTEM image (Figure 2g) of a selected area in Figure 2f reveals the base material foam-like morphology with several nanometer pore sizes and the layered arrangements of graphene. The most abundant element, carbon, is uniformly distributed throughout the sample, in contrast to copper and oxygen, which are expectedly distributed randomly in the material (Figure S3b–d). The density of the copper nanoparticles is extremely high here. The amounts of these three elements are summarized in Tables S1 and S2. This points out that the fabricated material is made of high-density metal-embedded graphene.

### 3.2. Electrochemical Feature

To shed light on the electrochemical features of the LIMG and Tyr/LIMG electrodes, cyclic voltammetry (CV) and differential pulse voltammetry (DPV) analyses are carried out using a portable smartphone-assisted electrochemical wireless USB-like platform, which is manufactured by Shenzhen Refresh Biosensing Technology Co., Ltd. (Shenzhen, China); device model: Biosys P15E MAX. Figure 2h shows the CV curves for [Fe(CN)_6_]^3−^/KCl redox probes. A comparison of Faradaic peak separation (∆Ep) and peak current ratio I_o/r_ = I_ox_/I_red_, shows that LIMG has a higher ability than Tyr/LIMG. As-fabricated LIMG exhibits such performance as a result of improvements in electroactivity, surface functionalities, and reaction kinetics [46]. However, when tyrosinase enzyme is added to the LIMG WE, the redox potential of [Fe(CN)_6_]^3−^/KCl is moderately reduced due to slow electrode kinetics provided by the enzyme’s nonconducting layer on the electrode surface, validating the presence of tyrosinase [9,26]. Moreover, the interface properties of the modified electrodes are characterized by the electrochemical impedance spectroscopy (EIS) study. As shown in Figure S4, the semicircle portion of the Nyquist plot is attributed to the charge transfer resistance (R_CT_) caused by the electron transfer of the redox probe [Fe(CN)_6_]^3-^. The R_CT_ value can be estimated to be 268 Ω and 628 Ω for bare LIMG and Tyr/LIMG, respectively. The higher R_CT_ value of Tyr/LIMG discloses that the electron transfer resistances were significantly increased by enzyme immobilization.

The DPV approach is employed to examine the precise sensitivity, specificity, and analytical capability of the LIMG, Tyr/LIMG, and commercial GCE against DA with probable interferences, as illustrated in Figure 2i. Tyr/LIMG has a very strong and clear DA peak (200 mV), in a combination including DA, ascorbic acid (AA), and uric acid (UA), with AA and UA concentrations 10 times greater than DA. These three peaks are well-differentiated anode current responses in the presence of potential interferents (1 mmol/L AA and 1 mmol/L UA) for detecting 0.1 mmol/L DA, which is attributable to AA, DA, and UA oxidation at potentials of 0.49 V, 0.2 V, and 0.348 V, respectively, which refers to the oxidation-reduction reactions in the following order [9].

1. Dopamine + Tyr(ox) → Dopamine-O-quinone + 2H^+^ + Tyr(red);
2. Tyr(red) + 2 [Fe(CN)_6_]^3−^ ↔ Tyr(ox) + 2 [Fe(CN)_6_]^4−^;
3. 2 [Fe(CN)_6_]^4−^ ↔ 2 [Fe(CN)_6_]^3−^ + 2e^−^.

In the case of bare LIMG and GCE, we found three and two wide peaks with lower current response, respectively, where the signals of each molecule overlapped, resulting in low selectivity and sensitivity. In this study, the CV results display that the Tyr/LIMG has a lower oxidation peak in the presence of [Fe(CN)_6_]^3−^ alone, compared to bare LIMG. On the contrary, Tyr/LIMG exhibits a two times higher oxidation current in the presence of DA along with interferents in the DPV study. It is well known that DA is an electroactive compound with redox peaks at the majority of electrodes including LIMG and GCE, whereas the tyrosinase enzyme in Tyr/LIMG functions as a biocatalyst, DA capturer, and an electron mediator, catalyzing the oxidation reaction of DA into dopamine-O-quinone and acting as an interface for the flow of electrons at the bioelectrode surface, causing an increase in oxidation current via the simultaneous reduction of tyrosinase and [Fe(CN)_6_]^3−^, followed by [Fe(CN)_6_]^4−^ oxidation. This is in response to the enzyme’s active binding sites and improved electron transfer between the active sites and the electrode, which allows for more sensitive detection of dopamine. This phenomenon is consistent with the previous works [9,55]. On the whole, the results of the DPV study demonstrated that the Tyr/LIMG electrochemical sensor has remarkable selectivity and sensitivity for detecting DA.

**Figure 3:**
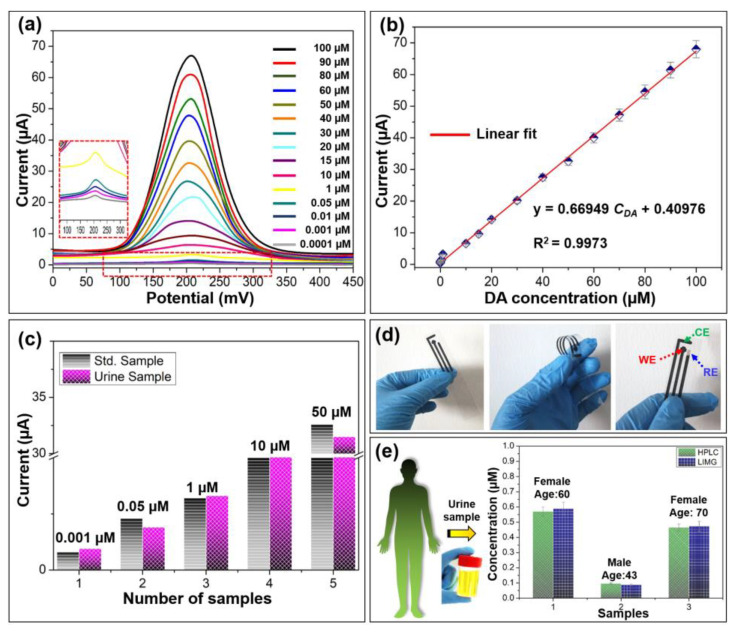
(**a**) DPV curves of the Tyr/LIMG modified sensor for various concentrations of dopamine (0.0001–100 µmol/L). (**b**) Calibration curve of current versus dopamine concentration. (**c**) Real-sample analysis results are plotted as current response versus both (standard and serum) samples. (**d**) Photographic images of the as-fabricated integrated sensor. (**e**) Real urine samples analysis by the HPLC method and the as-fabricated Tyr/LIMG sensor.

Further examining the sensitivity of the Tyr/LIMG sensor against DA, the DPV experiment to various concentrations of DA (0.0001–100 µmol/L) is conducted under constant stirring. The typical DPV curves of different DA concentrations, in relation to current response Vs potential, are shown in Figure 3a. At a potential of 200 mV, it is determined that the anodic peak current increased linearly with the increasing DA concentration. The as-fabricated Tyr/LIMG sensor displays an excellent linear range of detection (Figure 3b). The linear relationship between I_pa_ and C_DA_ is I_pa_ = 0.66949 C_DA_ + 0.40976, where I_pa_ is the current response in µA, and C_DA_ is the concentration of DA in µmol/L. The calculated value of the limit of detection (LOD) of this Tyr/LIMG sensor is 100 pmol/L (S/N = 3) with a sensitivity of 669.49 nA (µmol/L)^−1^ and a limit of quantification (LOQ) =337 µmol/L, which is an excellent LOD to determine the DA in real physiological samples (urine, serum, and saliva) [13].

### 3.3. Real-World Application Study

In addition, we investigated key aspects, such as real-sample and reliability analyses, to assess the commercial use of this sensor. To determine the practicality of this study, the constructed sensor is used to find DA in human urine samples spiked with known DA concentration. The strong correlation between the standard and real samples is shown in Figure 3c. It displays an acceptable relative standard deviation (RSD) of 2.6–3.8% and a decent recovery range of 92.6–100.2%. Figure 3d shows photographic images of the integrated sensor as fabricated, demonstrating its high processability and flexibility. Finally, the fabricated sensor is tested for real-world applications. It detects DA at the concentrations of 0.59, 0.088, and 0.473 μmol/L in the urine samples of the volunteers (Figure 3d). We additionally examined the same samples using the HPLC method to ensure the validity of the data obtained, which shows good agreement with the t-test value of 2.45 in 4° of freedom. The obtained statistical results are tabulated in Tables S3 and S4. It proves the applicability of possible clinical usage for DA detection noninvasively.

Moreover, to assess the practical application of this sensor, specificity, consistency, longevity, and volatility investigations are undertaken. The Tyr/LIMG sensor demonstrates good sensing performance when DA (40 µmol/L) is added, as shown in Figure S5a. There is no substantial response to the addition of the equimolar species of chemicals. The introduction of AA and UA, in particular, has no effect on the current amplitude of the Tyr/LIMG. This finding agrees with the results of DPV analysis, which further confirms the Tyr/LIMG composite’s outstanding specificity for DA detection. Five Tyr/LIMG sensors are made concurrently under identical circumstances. The same current response (RSD 4.6%) for all of the sensors shown in Figure S5b demonstrates the manufacturing method’s consistency. The Tyr/LIMG longevity test chart is shown in Figure S5c and it is examined on a five-day basis for 60 days. The material is durable for an extended time at typical storage circumstances since 97.2% of the current signal and operating function are kept intact. Furthermore, prolonged use of the Tyr/LIMG sensor for up to 50 cycles preserves 90.9% of the current signal, indicating the sensor’s outstanding durability and antifouling characteristic (Figure S5d). This performance significantly overtakes those reported recently (Table 1).

### 3.4. Material Formation and Sensing Mechanism

The results of the physicochemical analysis confirm that the LIMG is made up of graphene-foam structures and copper nanoparticles. The photoreduction process, which is associated with hydrolyzed PET, causes the copper ions to be reduced when the surface of precursor materials is exposed to suitable laser light at 473 nm. In this case, copper dithiocarbamate (CDC) has a strong absorbance at around 450 nm while PET is completely transparent. The drastic material transformation is relatively poor in the case where the wavelength of the incident light is almost at the edge of the CDC’s absorption band, i.e., hv–Eg. Low-single photon absorption may be the cause of this phenomenon. But, as exposure time and light intensity are increased, optically induced photothermal effects come in greater capacities [65]. As a result, the graphitization of the PET surface is accompanied by the formation of tiny copper particles. At the surface/interface of the precursor material, copper-ion reduction and PET graphitization both happen simultaneously with abundant oxygen functionalities [66].

**Figure 4:**
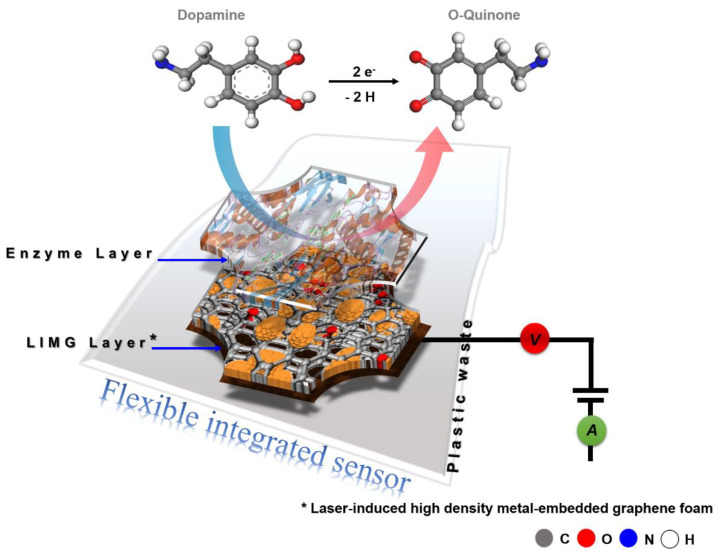
Schematic illustration of the materials-analyte-transduction mechanism.

The excellent performance of the developed sensor has been observed, as shown in Figure 3 and Figure S5. This distinct behavior can result from all of the components listed below: (1) Due to the synergy of copper nanoparticles and graphene foam, high-density metal-embedded graphene foam undergoes rapid charge transfer via electron coupling, resonance, and the surface plasmon effect during operation. (2) The interface layer of the porous structure facilitates the chemical’s quick passage through the foam. (3) Through an effective H-bond interaction, the immobilized enzyme molecule functions as both an effective biomolecule selector and a capturer. (4) The sensor surface’s net negative charge expels the UA, AA, and other negatively charged interferents. Schematic illustration of the whole materials-analyte-transduction process is presented in Figure 4.

## 4. Conclusions

A flexible highly efficient biosensor is developed by a one-step laser-scribing process of waste plastic PET to detect a DA noninvasively. Remarkable physicochemical properties are achieved when the Cu ion-treated waste PET sheet is exposed to the visible laser light under appropriate conditions, resulting in the formation of the laser-induced metal-embedded graphene-foam nanocomposite. The as-fabricated biosensor is evaluated against the detection of DA in a PBS/real-sample solution. It senses DA down to 100 pmol/L in the urine samples with the presence of interferents, which is the very much lower detection limit of the recently reported polymer–metal–carbon-based sensor (Table 1). Further results of the real-sample (disease-diagnosing human urine) analysis could prove the possible usage of portable fabricated POC devices for the clinical examination of DA. Nevertheless, large amounts of data remain needed to justify it, which provides a new avenue for advancement in the future. The current study adds to our understanding of how to develop an effective neurochemical sensor using a simple laser-scribing process of plastic waste, which is abundant and polluting our environment.

## Figures and Tables

**Table 1:** A comparison between the results of this study and previously published data utilizing different sensors for DA sensing.

Carbon/Polymer/Metal-Based Sensing Probe	Linear Range (µmol/L)	LOD (mol/L)	Real Sample Invasive/Noninvasive	Ref
**rGO-PAP**	0.001–100	2.0 × 10^−9^	Invasive	[10,13]
**rGO-PAS**	0.001–100	1.1 × 10^−10^	Invasive	[13]
**rGO-PAB**	0.0001–100	1.0 × 10^−11^	Invasive	[13]
**Au/PPy/rGO**	0.001–5	1.8 × 10^−11^	Invasive	[14]
**Carbon NRs**	1–10	6.0 × 10^−8^	Invasive	[27]
**POMF-rGO**	1–200	8.0 × 10^−8^	NA	[28]
**HOPG-β-cyclodextrin**	-	1.0 × 10^−7^	NA	[29]
**NACP**	0.05–15	1.0 × 10^−8^	Invasive	[30]
**MIP/MWCNT/GAs/GCE**	0.005–20	1.6 × 10^−6^	Invasive	[31]
**SPANI/CNSs/GCE**	0.5–1780	1.5 × 10^−8^	NA	[32]
**Ni-MOF**	0.2–100	6.0 × 10^−8^	Invasive	[34]
**PPy/ZIF-67/Nafion**	0.08–100	3.0 × 10^−8^	Invasive	[35]
**Ni_3_HHTP_2_-MOF**	0.04–200	6.3 × 10^−8^	NA	[37]
**rGO-Au NPs/ITO**	0.02–200	1.5 × 10^−8^	Invasive	[38]
**rGO-SS**	1–1000	1.0 × 10^−6^	NA	[40]
**TiN-rGO**	5–175	1.59 × 10^−7^	Noninvasive	[41]
**rGO-PTA**	0.5–20	-	NA	[42]
**rGO-MWNT-PTA**	0.5–20	1.14 × 10^−9^	NA	[43]
**rGO-Pt**	10–170	2.50 × 10^−7^	NA	[60]
**rGO-Pd NPs**	1–150	2.33 × 10^−7^	Invasive	[61]
**3D SWNT–PPy composite**	5–50	5.0 × 10^−6^	NA	[62]
**rGO-GC**	0.1–100	1.0 × 10^−7^	NA	[63]
**Carbon fiber**	-	4.1 × 10^−8^	NA	[64]
**Tyr/LIMG**	**0.0001–100**	**1.0 × 10^−10^**	**Noninvasive**	**This work**

NA: Not applicable, NP: Nanoparticle, SS: Stainless steel, NT: Nanotube, NR: Nanorod, PTA: Phospotungstic acid, PPy: Polypropylene, MWCNT: Multiwall Carbon nanotube, GC: Glassy carbon, POMF: Polyoxometalate-based metal–organic framework, HOPG: Highly oriented pyrolytic graphite, MOF: Metal–organic framework, NACP: Ni-MOF composite/AuNPs/CNTs/PDMS, SPANI: sulfonated polyaniline, CNS: Carbon nanospheres, G: Graphene, GCE: Glassy carbon electrode.

## Data Availability

The data that support the findings of this study are available from the corresponding author upon reasonable request.

## Supplementary Materials

The following supporting information can be downloaded at: https://www.mdpi.com/article/10.3390/bios13080810/s1, Information S1: Experimental details; Figure S1: UV-Vis spectra of precursor sample and laser light; Figure S2: Copper nanoparticle size calculation with Gaussian peak fit; Figure S3: (a) HAADF image of Figure 2f (main text). Corresponding Energy-dispersive X-ray spectroscopy mapping images of (b) Copper, (c) Carbon, and (d) Oxygen; Figure S4: Electrochemical impedance spectra of bare LIMG and Tyr/LIMG-modified electrodes in an [Fe(CN)_6_]^3−^/PBS solution; Figure S5: (a) Amperometry specificity study of the Tyr/LIMG and electrocatalytic oxidation of DA (40 µmol/L) along with equimolar EP, 5HT, AA/UA, K^+^Cl^−^, Ca^2+^, Mg^2+^, glucose, and glutamic acid, operated at +200 mV in PBS. (b) The current responses of five parallelly fabricated Tyr/LIMG sensors toward DA (40 µmol/L) (n = 3). (c) Change in current response against the number of days towards DA detection (n = 3). (d) Subsequent measurement of Tyr/LIGM in the 40 µmol/L DA/PBS, recorded for 50 cycles by reusing the same sensor; Figure S6: FT-IR spectra of the PET sheet cut from a PET bottle, which is recorded from three different bottles. Inset: Molecular structure of PET consists of C, H, and O as the major species; Table S1: Chemical species data in at% obtained from EDS measurement; Table S2: Chemical species data in Wt% obtained from EDS measurement; Table S3: Detection of DA spiked in urine samples; Table S4: Detection of DA in human subjects’ urine samples [67,68].
