# Supplementary material for: Laser Scribing Turns Plastic Waste into a Biosensor via the Restructuration of Nanocarbon Composites for Noninvasive Dopamine Detection
Source: Biosensors (Basel). 2023 Aug 12;13(8):810. doi: 10.3390/bios13080810 (PMC10452382; doi:10.3390/bios13080810)
Supplement: Supplementary file 1 [file biosensors-13-00810-s001.zip › biosensors-2505862-supplementary.pdf]

Supporting Information

Laser Scribing Turns Plastic Waste into a Biosensor via the Restructuration of Nanocarbon Composites for Noninvasive Dopamine Detection

Jagadeesh Suriyaprakash [1], Yang Huang [1,†], Zhifei Hu [1,†], Hao Wang [1], Yiyu Zhan [1], Yangtao Zhou [2], Indumathi T [3] and Lijun Wu [1,*]

Supporting Information contains

1. Experimental details
2. Figures S1-S6
3. Table S1-S4

1. METHODS

1.1. Materials

From the Sigma-Aldrich company, various chemicals were purchased, including Dopamine (DA), epinephrine (EP), norepinephrine (NEP), serotonin (5-HT) (>99%), and lyophilized tyrosinase powder (⩾1000 units/mg). From the Merck company, uric acid (UA), ascorbic acid (AA), glucose (G), glutamic acid (GA), sulphuric acid, PAB, methanol, ethanol, dichloromethane (DCM), potassium hexacyanoferrate(III) ($K_3Fe(CN)_6$), potassium chloride (KCl), ethanolamine, 1-ethyl-3- (3-dimethyl aminopropyl) carbodiimide-N-hydroxysuccinimide, and copper dithiocarbamate were acquired. All solution preparations and dilutions of the samples were carried out in MilliQ water (18.2 MΩ·cm). From Aladdin Co., Ltd, the phosphate-buffered saline (PBS) solution with PH 7.2 was used as a supporting electrolyte containing KH_2PO_4 and K_2HPO_4. Unless otherwise specified, all solvents and chemicals used in this investigation were of analytical grade and utilized exactly as provided.

1.2. Characterization

A Rigaku D/max 2200V/PC diffractometer with Cu Ka (λ = 1.54178 Å) was utilized to obtain the X-ray diffraction pattern (XRD) of the samples. In backscattering mode, the Lab Ram Horiba-Jobin Yvon spectrometer was used for Raman spectroscopy measurements. At an exposition duration of 5 seconds, the spectrum was obtained. The 1st-order Raman spectrum of silicon monocrystalline was analyzed before and after the acquisition of each Raman spectrum and the spectra were calibrated with respect to the silicon peak location at 520 cm^{-1}. X-ray photoelectron spectroscopy (XPS) using a Thermo Electron Corporation ESCA Lab250 spectrometer at 15kV and 150 W was employed to study the surface characteristic of the synthesized samples. The high-resolution transmission electron microscopic (HRTEM) and energy-dispersive X-ray spectroscopy were carried out in an FEI Tecnai G2 F30 microscope in which an electron beam is generated from the field emission gun at 300 KeV.

1.3. Electrochemical study

A handheld electrochemical device (REFRESH-Biosys) was used for all of the electrochemical investigations unless mentioned. A glassy carbon electrode (GCE) was also used as WE as a means of evaluation. By scanning a voltage range between -0.9 V and +0.9 V vs. Ag/AgCl and at a scan rate of 100 mVs^{-1} and pH value of ~7.2 in 3mmol/L $[Fe(CN)_6]^{3-}$/PBS solution, cyclic voltammograms were produced. Additionally, the interface properties of the modified electrodes are characterized by the electrochemical impedance spectroscopy (EIS) study (operate at 10^{-1} to 10^5 Hz at an amplitude of 5 mV) using an electrochemical workstation (Autolab, PGSTAT302N).

By scanning the potential between -0.05 V and 0.50 V, with a pulse height of 100 mV, a pulse time of 0.4 s, a voltage step time of 0.5 s, and a voltage step height of 1 mV, differential pulse voltammograms (DPV) were acquired.

For real-sample analysis, human urine was used to evaluate the performance of the LIMG and Tyr/LIMG electrodes toward DA. The samples were prepared as follows; the entire study protocol was followed as per the ethics committee's guideline of "SCNU University". Three volunteers' urine samples (those who were diagnosed with Parkinson's disease) were collected with mutual understanding and agreement. For the normal (healthy people) urine sample study, JS (the first author) volunteered. First, the obtained urine was centrifuged to remove the precipitates and solid impurities. Next, the urine samples were diluted 10 times with 3 mmol/L of $K_3Fe(CN)_6$/KCl solution. Then, the diluted urine samples were utilized for further study. For standard and real sample comparison analysis, diluted urine was spiked with a known amount of DA; various concentrations were tested (0.001, 0.05, 1, 10, and 50 μmol/L). All the electrochemical studies were conducted at the pH value of ~7.2.

1.4. Processing of data

As shown below, we followed the established procedure to analyze the data gathered in order to compute further metrics.

1. The limit of detection (LOD) at lower concentrations of analyte is based on the least detectable signal (Signal/Noise = 3, 95% statistical confidence level);
2. Limit of quantification= 10*σ / S (where S is the slope, σ is std.dev of net quantity);
3. Relative standard deviation (%) for three data sets;
4. t-test=paired t-test at the 95% confidence level;
5. For the electrochemical examination, after eradicating the noise, DPV curves were drawn using a spline fit by picking locations along the curve both before and after the

potential range matching to the faradaic peak. The Linear fit curve was produced using OriginPro software on the entire data set.

6. The error bars for the electrochemical results represent the standard error of the mean and were calculated after evaluating three distinct devices (n = 3) for every operating step.

2. RESULTS

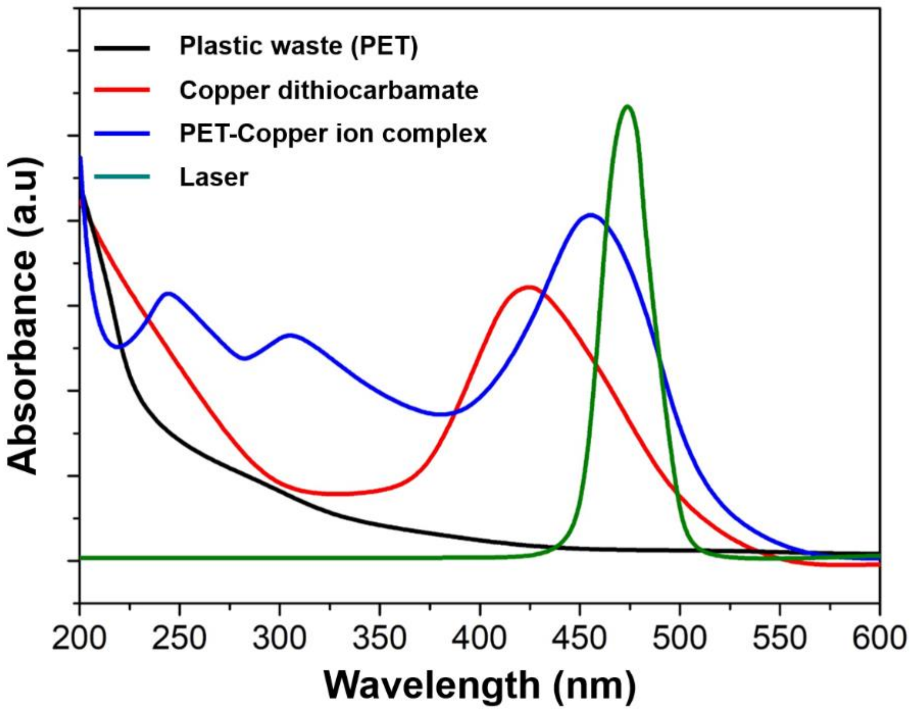

Figure S1. UV-Vis spectra of precursor sample and laser light.

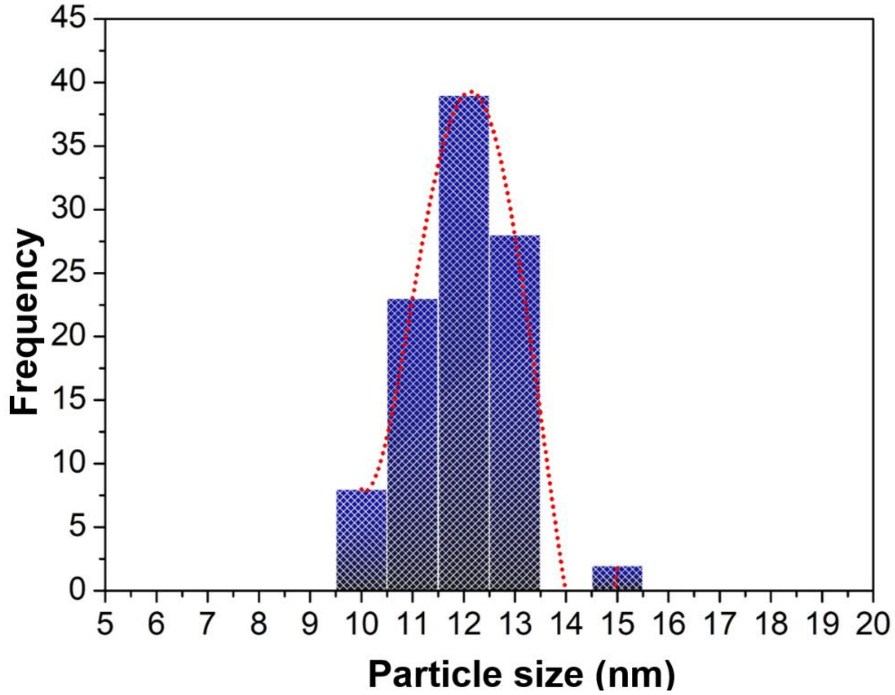

Figure S2. Copper nanoparticle size calculation with Gaussian peak fit.

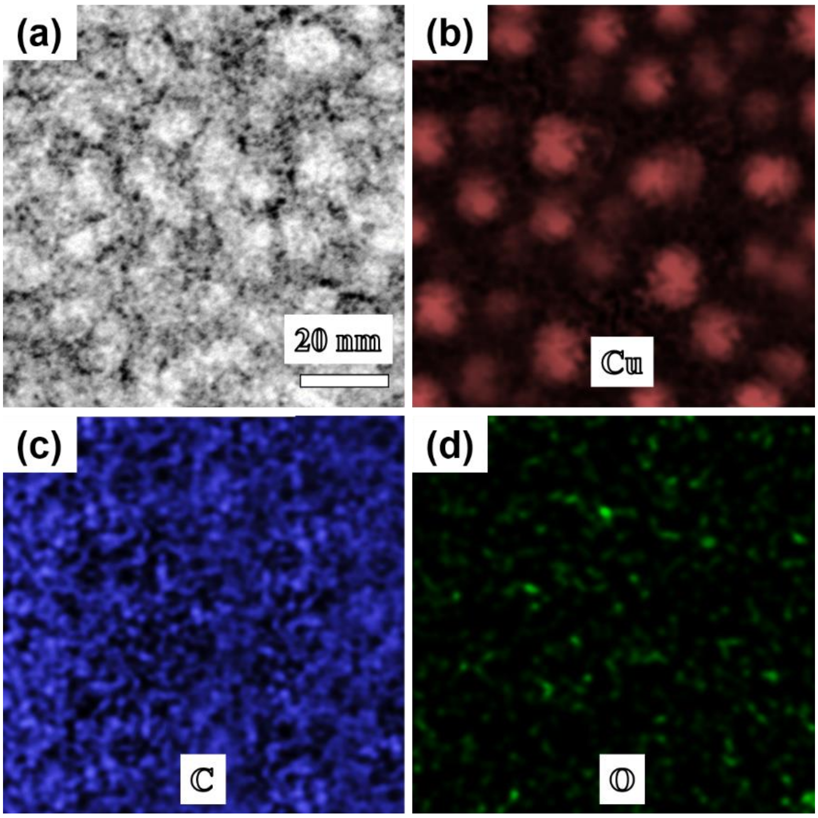

Figure S3. (a) HAADF image of Fig. 2f (main text). Corresponding Energy-dispersive X-ray spectroscopy mapping images of (b) Copper, (c) Carbon, and (d) Oxygen.

Table S1. Chemical species data in at% obtained from EDS measurement

Sample	C	Cu	O	S
**LIMG**	62.72	28.72	7.21	1.35

Table S2. Chemical species data in Wt% obtained from EDS measurement

Sample	C	Cu	O	S
**LIMG**	44.61	51.76	1.12	2.51

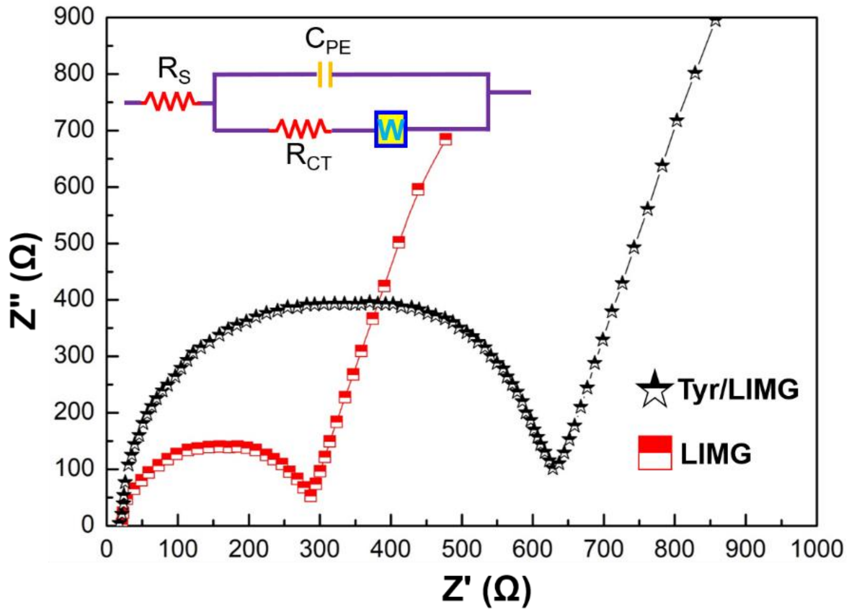

Figure S4. Electrochemical impedance spectra of bare LIMG and Tyr/LIMG modified electrodes in an $[Fe(CN)_6]^{3-}$/PBS solution.

EIS measurement in 3mM of $[Fe(CN)_6]^{3-}$ solution was performed to further understand the electron transfer kinetics in LIMG and Tyr/LIMG electrodes. Nyquist plots (Fig. S4) of imaginary (Z") versus real (Z') parts were obtained. The circuit is made up of the R_S component, which is the ohmic resistance from the solution, in series with the constant phase element (CPE) parallelly combined with the charge transfer resistance (R_{CT}) of the Faradaic process and the Warburg impedance (W) component arising from mass diffusion towards the electrode surface. Because of the heterogeneity in the electrode surface, which contributes to the imperfect capacitance, the CPE component is used instead of the true double-layer capacitance (C_{dl}). The

R_{CT} values obtained for the Faradaic process occurring at various modified electrodes are 268 Ω and 628 Ω for bare LIMG and Tyr/LIMG, respectively.

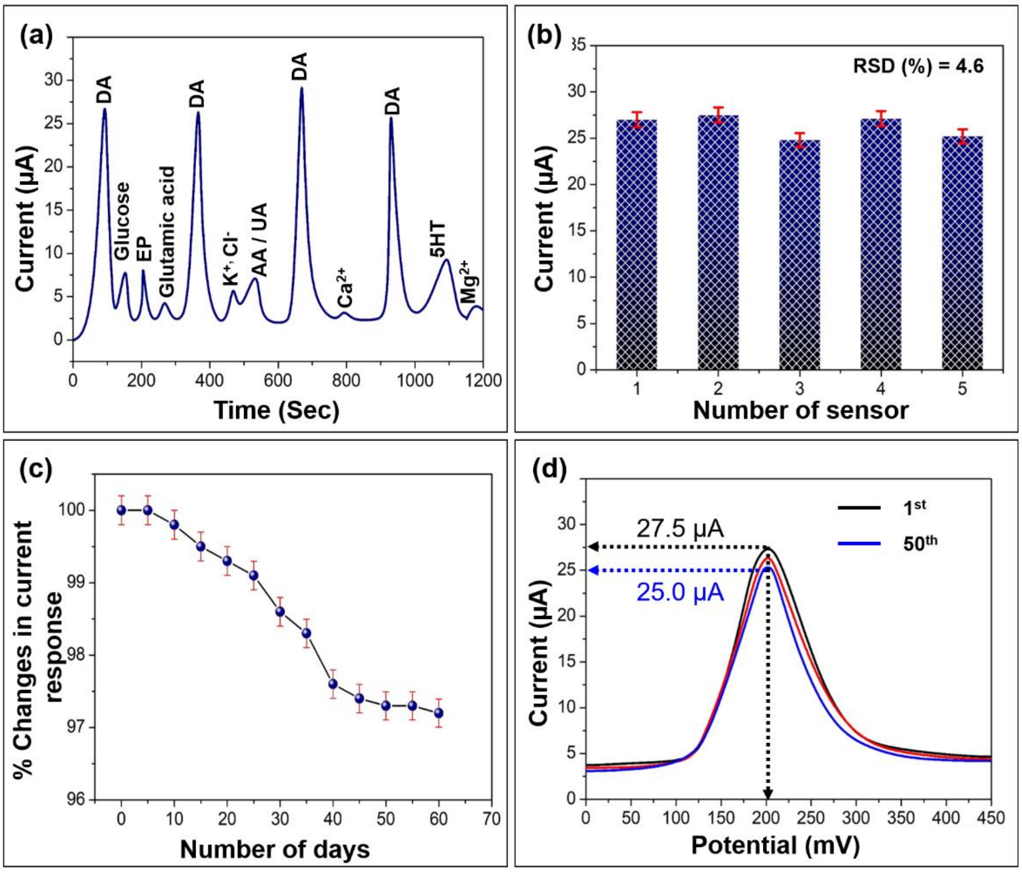

Figure S5. (a) Amperometry specificity study of the Tyr/LIMG and electrocatalytic oxidation of DA (40 μmol/L), along with equimolar EP, 5HT, AA/UA, $K^{+}Cl^{-}$, Ca^{2+}, Mg^{2+}, glucose, and glutamic acid, operated at +200 mV in PBS. (b) The current responses of five parallelly fabricated Tyr/LIMG sensors toward DA (40 μmol/L) (n=3). (c) Change in current response against the number of days towards DA detection (n=3). (d) Subsequent measurement of Tyr/LIGM in the 40 μmol/L DA/PBS recorded for 50 cycles by reusing the same sensor.

Table S3. Detection of DA spiked in urine samples

Human urine	Spiked (µmol/L)	Found (µmol/L)	Recovery (%)	RSD (n=3) (%)
**Sample-1**	0.001	0.0011	100.2	2.8
**Sample-2**	0.05	0.0463	92.6	2.7
**Sample-3**	1	1.0312	100.3	3.2
**Sample-4**	10	9.234	92.3	2.6
**Sample-5**	50	48.312	96.6	3.8

Table S4. Detection of DA in human subjects' urine samples

Human urine	This work sensor (µmol/L)	RSD (n=3) (%)	Reference HPLC method (µmol/L)	RSD (n=3) (%)	t-Test[a]
**Subject-1**	0.59	1.9	0.57	0.5	2.45
**Subject-2**	0.088	0.9	0.096	1.2	1.81
**Subject-3**	0.473	1.1	0.465	0.7	1.52

[a] Tabulated t–value for 4° of freedom at a P value of 0.05 is 2.58.

The obtained results for the detection of DA in human urine samples using the proposed Tyr/LIMG were compared with the reference values obtained by the HPLC method by applying the statistical calculations (paired t-test). At the 95% confidence level, the calculated (experimental) values of the paired t-test are less than their tabulated (theoretical) values, implying that there was no noticeable difference between the performances of the compared methods regarding precision and accuracy.

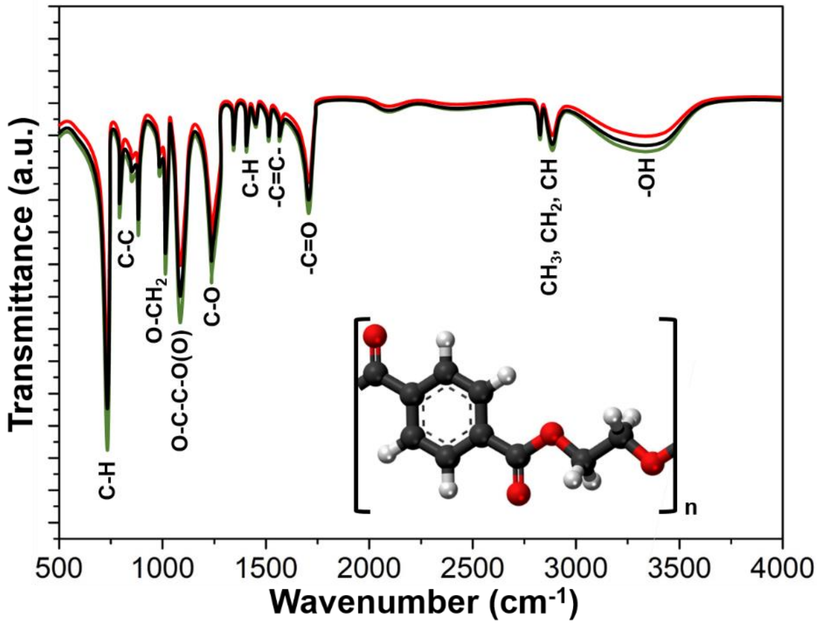

Figure S6. FT-IR spectra of the PET sheet cut from PET bottle, which is recorded from three different bottles. Inset: Molecular structure of PET consists of C, H, and O as the major species.

The chemical composition of the waste PET bottles is subjected to FTIR spectral analysis to ensure the reliability of the source material (Fig. S6). Three different sources are investigated. Characteristic CH, C-C, O-CH_2, O-C-C-O(O), C=O, and OH vibrational peaks, as well as fingerprint regions, are observed in Fig. S6, demonstrating that the obtained PET sheet from waste plastic has a major composition of C, H, and O in the form of PET polymer as shown in Fig. S6 inset. The observed spectra results agree well with previous work [67,68 in main text]. As a result, the precursor material used for this study contains PET as a high majority.
